# Intra-Articular Injections of Platelet-Rich Plasma versus Hyaluronic Acid in the Treatment of Osteoarthritic Knee Pain: A Randomized Clinical Trial in the Context of the Spanish National Health Care System

**DOI:** 10.3390/ijms17071064

**Published:** 2016-07-02

**Authors:** Elvira Montañez-Heredia, Sofia Irízar, Pedro J. Huertas, Esperanza Otero, Marta del Valle, Isidro Prat, Macarena S. Díaz-Gallardo, Macarena Perán, Juan A. Marchal, María del Carmen Hernandez-Lamas

**Affiliations:** 1 Department of Orthopedic Surgery and Traumatology, Virgen de la Victoria University Hospital, Málaga E-29010, Spain; sofiairizar@gmail.com (S.I.); pedrojhuertascot@hotmail.com (P.J.H.); 2 Department of Anestesiology. Pain’s Treatment Unit. Virgen de la Victoria University Hospital, MálagaE-29010, Spain; espeotero@hotmail.com (E.O.); martadelvalle75@hotmail.com (M.d.V.); 3 Málaga Tissue Bank, Regional Blood Center, Málaga E-29009, Spain; isidro.prat.sspa@juntadeandalucia.es (I.P.); macarenas.diaz.sspa@juntadeandalucia.es (M.S.D.-G.); carmen.hernandez.lamas.sspa@juntadeandalucia.es (M.d.C.H.-L.); 4 Department of Health Sciences, University of Jaén, Jaén E-23071, Spain; mperan@ujaen.es; 5 Biopathology and Regenerative Medicine Institute (IBIMER), Centre for Biomedical Research, University of Granada, Granada E-18071, Spain; jmarchal@ugr.es; 6 Department of Human Anatomy and Embryology, Faculty of Medicine, University of Granada, Granada E-18016, Spain; 7 Biosanitary Institute of Granada (ibs. GRANADA), University Hospitals of Granada-University of Granada, Granada, Granada E-18016, Spain

**Keywords:** platelet-rich plasma, knee osteoarthritis, clinical trial, hyaluronic acid

## Abstract

Intra-articular injection of platelet-rich plasma (PRP) has been established as a suitable treatment for knee osteoarthritis. Here, we present a double-blind randomized controlled clinical trial, conducted in a public Hospital of the Spanish National Health Care System, to evaluate the efficacy of injecting autologous PRP versus hyaluronic acid (HA) in knee osteoarthritis. PRP was manufactured in Malaga’s Regional Blood Center (Spain). Patients that met the eligibility criteria were randomized into a PRP group or a HA group. Pain and functional improvements were assessed pre- and post-treatment (three and six months follow-up) using the Visual Analogue Scale (VAS); the Knee and Osteoarthritis Outcome System (KOOS) scale and the European Quality of Life scale (EUROQOL). Both groups presented pain reduction at six months. The VAS scores for the PRP group improved by at least 50% from their initial value, particularly at three months following the final infiltration, with results resembling those of the HA group at six months. PRP was more effective in patients with lower osteoarthritis grades. Both treatments improved pain in knee osteoarthritis patients without statistically significant differences between them. However, PRP injection was proved to improve pain three months after the final infiltration and to be more effective in lower osteoarthritis grades.

## 1. Introduction

The increase in average life expectancy and the high incidence of knee arthritis has led to a search for less aggressive alternatives to joint replacement with a lower financial impact on the health system. Some novel strategies are based on stimulation methods for cartilage repair or for the inhibition of catabolic enzymes, gene therapy, artificial replacement cartilage and the use of growth factors.

Platelet-rich plasma (PRP) is a natural concentrate of autologous growth factors obtained through centrifugation of a patient’s own blood. PRP is obtained at a low cost and in a simple and minimally invasive manner using an “open technique” in blood banks or through a “closed technique” using disposable commercial kits. Bioactive cytokines and proteins from the platelet’s alpha granules induce chemotaxis, cellular migration, proliferation, differentiation and extracellular matrix production [1]. In addition, these proteins increase the release of angiogenic growth factors [2,3] contributing to tissue regeneration and cicatrisation [4]. The main growth factors contained in PRP are platelet-derived growth factor (PDGF), transforming growth factor β (TGF β), insulin-like growth factor (IGF-1) and fibroblast growth factor (FGF). Interestingly, these factors have been proved to be involved in chondrogenesis and cartilage regeneration [5,6].

Local infiltration of PRP, extensively studied in maxillofacial pathology [7,8], has achieved good results in soft tissue injuries [9,10]. Although the regenerating potential and role of PRP in stabilizing angiogenesis in arthritic knees has been recognized [2,11], its use in articular pathology has not yet been sufficiently supported by controlled studies. In fact, the hypothesis that PRP reduces pain has been argued by several studies [12,13,14]. Nevertheless, when pain persists after oral anti-inflammatory or analgesic medications the intra-articular injections of corticosteroids (CS), hyaluronic acid (HA) and platelet-rich plasma (PRP) is indicated before a surgical treatment is performed [15,16]. A revision of the conducted clinical trials evidence that intra acicular injections of HA may be useful in patients with knee osteoarthritis (OA). They are characterised by delayed onset, but prolonged duration of symptomatic benefit, such as improvements in pain on weight bearing, when compared to injections of corticosteroids [16]. A recent clinical study conducted by Sánchez and Anitúa et al. [17] concluded that PRP manufactured in a closed system designed and commercialized by the authors was shown to be more efficient in controlling pain than HA treatment.

The Spanish Agency of Medicines and Medical Devices (AEMPS) [18] has considered that the product known as PRP, obtained by blood centrifugation, is neither a hemo-derivative nor a biological product, but a medicine for human use. As such, PRP must be manufactured and supplied. AEMPS established that obtaining and processing this product must follow the standard quality-control measures for blood-derived products. Some authors indicate the need of more independent, prospective, randomized and controlled studies before PRP infiltration can be accepted into current practice [19,20,21]. In this respect, we present the first clinical trial performed under the directives of the AEMPS to test the efficacy of PRP infiltration to treat arthritic knees.

The initial hypothesis of the present study was that patients would get improved OA treatments if a close relationship between two health public centres were established. To test so, the main objective of the present study was to determine the efficacy of a clinic protocol developed in public healthcare centres that allow patients to benefit from the analgesic power of their own plasma, obtained from just a single puncture, processed in accordance with the safety regulations established by AEMPS.

## 2. Results

**Figure 1:**
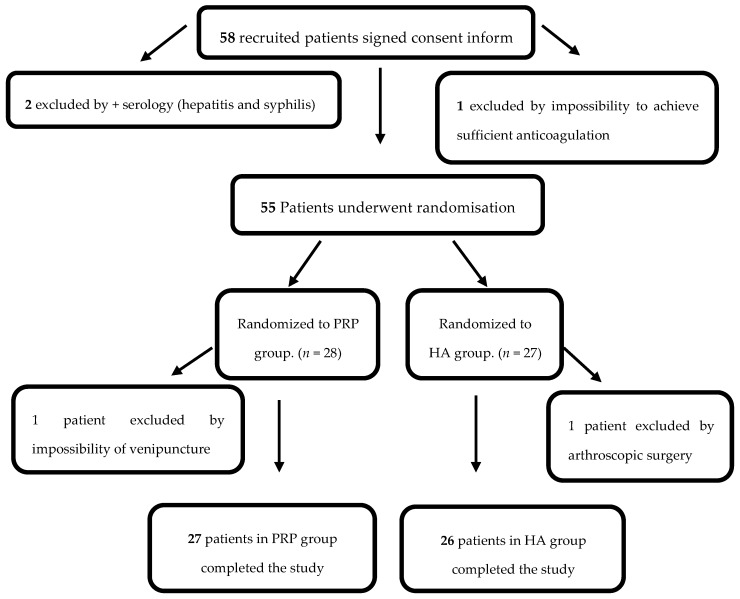
Flow diagram including the number of patients recruited, excluded and randomized at different times during the trial.

Based on the selection criteria (Table 1), 55 patients were chosen for this study and later randomized into a PRP group (*n* = 28) (group I) or a HA group (*n* = 27) (group II). One patient from group I was excluded by impossibility of venepuncture and one patient from group II needed arthroscopic surgery, and thus 27 patients in group I and 26 patients in group II completed the trial (Figure 1).

Table 2 shows the baseline characteristics of all patients where no differences were found in the parameters measured between groups. Briefly, there were 21 males (39.6%) and 32 females (60.4%), with an average age of 63.9 years (±8.8) and body mass index of 29.9 (±4.6). In 25 of 53 cases (47.2%), patients had pain in both knees, although they experienced more severe pain in the treated knee. For 28 of 53 patients (52.8%), symptoms were unilateral. The radiological osteoarthritis, measured using the Kellgren–Lawrence scale, was grade I in seven of 53 patients (13.3%), grade II in 19 of 53 patients (35.9%) and grade III in 27 of 53 patients (50.9%). The distribution of these variables in both groups was similar and, as such, the samples were considered homogenous.

PRP group patients were injected with PRP rich in platelets and weak in leukocytes and red blood cells, the composition of PRP is shown in Table 3. In order to test the platelet growth factors content of the PRP injections and the safety of PRP freezing and thawing cycle, patients PRP samples were analysed for growth factor concentration: hepatocyte growth factor (HGF), epidermal growth factor (EGF), fibroblastic growth factor-2 (FGF-2) and platelet-derived growth factor (PDGF) (Table 4). Although growth factor concentration varied from patient to patient, results showed that the concentration of growth factors of the PRP injections used in this study met requirements of commercials platelet concentrate used for transfusion therapy, such as MagellanPRP^TM^ (Global Orthobiologic, Miami, Florida, USA). Only two samples showed a low concentration of FGF-2, which could be due to methodological issues because the concentration of the other three growth factors in the same samples was normal.

Patients were clinically evaluated before the treatment, and at three and six months after the end of the treatment. Adverse events relating to infiltration were infrequent, mild and appeared immediately, and their distribution between both groups did not show significant differences. There was pain related to infiltration in nine of 27 PRP injections and in four of 26 for HA, but only one patient (in PRP group) had transitory swelling that resolved itself. No relationship between these events and the growth factor or blood cell composition of PRP was found.

Improvements were indicated on all of the pain scales used in this trial after treatment with both HA and with PRP. The knee injury and osteoarthritis outcome score (KOOS) symptoms subscale, excluding function in sport and recreation, showed improvement throughout treatment in both groups. Although, no statistically significant differences between groups were found at either three or six months after treatment, the symptoms subscale showed a tendency toward improvement in the PRP group after six months (*p* = 0.068). No differences were found in KOOS scale scores between patients with bilateral or unilateral symptoms. For patients with arthritis grade II, daily activities (ADL) at three months follow-up improved significantly on the KOOS scale in the PRP group as compared to the HA group (*p* = 0.040). At six months follow-up, pain decreased for arthritis grade II patients injected with PRP (*p* = 0.012) with improvements in function in daily living (*p* = 0.013) and function in sport and recreation (*p* = 0.021). On the other hand, in patients with arthritis grade III, no KOOS differences between PRP and HA groups were observed.

On the euro quality of life (EUROQOL) scale, patients injected with PRP improve mobility scores after three months from treatment when compared with HA group (*p* = 0.15), and the results were maintained, though not in a statistically significant manner, after six months follow-up (*p* = 0.9). Daily activities at three months follow-up improved slightly more in PRP group than in the HA group, nine of 27 patients in the PRP group versus three of 26 patients in the HA group (*p* = 0.14), and these differences in improvement were maintained at six months (seven of 27 versus three of 26), though without reaching statistical significance (*p =* 0.47). Additionally, personal care was maintained or improved at three months follow-up in more patients in PRP group when compared with hyaluronic acid HA group (25 of 27 versus 17 of 26) (*p =* 0.13). Similar results were obtained at six months follow-up (22 of 27 versus 18 of 26). On the pain subscale, positive evolution was noted in the PRP group, although not significantly, with more patients reporting improvements in pain at three and six months follow-ups (*p* = 0.44 and *p =* 0.63, respectively). Only two of 27 patients in the PRP group and four of 26 patients in the HA group experimented an increase in pain at six months follow-up from treatment with respect to the basal value (Table 5).

On the contrary, control group patients, treated with oral therapies, did not show improvements in the health related quality of life. After six months of treatment, 56% to 66% of the patients had worsened his condition, and 43% to 33% had not improved it at all (Table 6).

After six months from treatment, the VAS value improved in both groups (*p =* 0.001). It should be noted that, at three months follow-up, the PRP group experienced a reduction of at least 50% of the initial VAS value that was superior to the pain reduction experimented by patients treated with HA, although with no statistical significance (*p =* 0.22) (Table 7). Acetaminophen was required by some patients with no significant differences between the PRP and AH groups (*p =* 0.78).

In the control group (patients treated with oral therapies), only 10 patients (33.3%) showed a decrease of 50% in VAS score when compared with the baseline, after three months of treatment. This result worsened even more after six months of treatment with only three (10%) patients maintaining the 50% VAS score reduction.

Knee arthritic patients suffer from a high prevalence of pain, specifically of the knee, that have to be managed by chronic pain treatment units. In the present study, 36 patients were treated by orthopedics and 17 patients were treated by anesthetists. There were no differences between patients selected and treated by either specialist, with similar end results reported on all scales six months after treatment (Table 8).

## 3. Discussion

PRP have emerged as an alternative treatment of severe knee osteoarthritis. Here, we have tested the effectiveness of PRP injections to treat osteoarthritis patients in the context of the public health care system. Whole blood was extracted from the patients and PRP was processed, analysed and stored by a public transfusion centre and the surgery intervention was performed by the orthopedic service of a public hospital. Others studies have shown the benefits of PRP injections, demonstrating that the treatment improves pain control when compared with placebo [17,21,22]. In the present trial, HA injection was used as control treatment in agreement with previous clinical studies [17,23,24,25].

The selection criteria were established based on similar studies. Specifically, patients with Kellgren–Lawrence grade IV osteoarthritis were excluded because it has been proved that, in such cases, the intra-articular non-cortisone treatment is less effective (19). Just one patient was lost from group II (HA) once the study was underway, which represented a loss rate of only 1.8%, significantly lower than in previous studies [21].

Following AEMPS recommendations, serology testing for infectious diseases was performed prior to the study preventing antigenic contamination by syphilis in one patient and by hepatitis in other. The data on serology testing reinforces the importance of carrying out serology prior to a study.

Some authors have questioned the suitability of the use of frozen samples of PRP, due to a possible reduction of growth factor concentration [26]. However, several clinical trials have been performed using frozen PRP and have reported clinical improvement in knee osteoarthritis patients [13,23,27,28]. In agreement, other studies support the idea that freezing does not alter the beneficial effects of PRP on chondrocytes and synoviocytes [29]. In the present study, we demonstrate that stored autologous PRP frozen samples seem to maintain the necessary growth factors for promoting a beneficial effect on pain decrease in osteoarthritis patients. The concentration of growth factors of the PRP injections used in this study met requirements of prepared platelet concentrate used for transfusion therapy [26].

Here, we used PRP rich in platelets and poor in leukocytes, in agreement with previous studies [30]; however, the ideal leukocyte content is still under debate and it appears that, depending on the pathology, a higher concentration of leukocytes might be beneficial [31]. Some studies have even reported favourable clinical outcomes even with lower platelet concentrations [23,32,33], but variability in the secretion of platelet granules among patients, and even at different times in the same patient, make it difficult to compare the results obtained. We want to emphasize that, in our study, PRP for each patient was produced after a single extraction, and the three administered doses were identical in composition, reducing variability between PRP injections. Our system, when compared with the use of commercial kits, minimizes the risk of contamination and the risks related to the repeated venipuncture processes performed on the patient. In summary, PRP was obtained by a single extraction and manipulated and stored in installations that met quality and safety standards guaranteed by a homologated blood transfusion center (Regional Blood Center, Málaga, Spain).

A six-month period was established as the end point for testing because it has been shown that the benefits provided by the intra-articular injection of PRP appears to be reduced after six months from treatment [34]. The results of patients’ evaluations demonstrate that an improvement on all clinical scores tested was obtained from the basal evaluation, to the three and six month follow-ups following both treatments; on the other hand, patients treated with oral therapies did not show amelioration. The KOOS symptoms subscale showed that PRP injections were more effective than HA injections in patients with grade II arthritis, but not as effective in patients with grade III arthritis. In agreement with the present work, recent studies have shown that PRP is as effective as HA in the treatment of knee arthritis, showing more effectiveness for patients with lower grades of arthritis [20,35]. On the EUROQOL scale, scores improved in both treatments after six months, with a slight, although not significant, increment in favourable recovery in the PRP group compared with the HL group. Furthermore, patients in the PRP group experienced a reduction of at least 50% of their initial VAS pain levels. Others authors have reported that patients treated with PRP experimented the same reduction (50%) on the VAS scale but only after six months from treatment [17], this discrepancy with our data could be explained by the difference in the number of patients enrolled in each study.

Previous studies that include basic research and preclinical and clinical trials have produced evidence of the beneficial effects of PRP treatment for OA patients. A thorough review of those research findings made by Xie et al. [36] revealed the anti-inflammatory potential of PRP, its anabolic effect on chondrocytes mesenchymal stem cells (MSCs) and synoviocytes, and even its possible role in cartilage regeneration acting as a bioactive cell scaffold.

Although clinical evidence has favoured PRP over HA for treatment of OA, the viscose nature of HA and its proven beneficial effect in reducing pain and improving viscoelasticity of synovial fluid cannot be underestimated. In fact, the active anti-inflammatory or chondroprotective effect of HA has been reported [37]. In this context, a recent relevant study has proposed the combined use of PRP and HA, demonstrating that PRP addition is not detrimental to the viscosupplementation effect of HA [38].

Finally, we would like to state that circuits have been developed in public healthcare centres that allow patients to benefit from the analgesic power of their own plasma, obtained from just a single puncture (with the same composition) and processed in accordance with the safety regulations established by AEMPS. This method improves PRP obtained from commercial centrifugation systems, which require a puncture each time and provide a different composition in each infiltration, depending on the haematological status of the patient.

Further studies are needed to improve medical evidence available and to allow for the compilation of a specific technical dossier before PRP can be accepted into current practice.

## 4. Experimental Section

### 4.1. Interventional Study

The present clinical trial was registered as EUDRACT: 2013-001303-36 in European Clinical Trials Database and as Identifier: NCT02448407 in US ClinicalTrials.gov.

The full trial protocol can be accessed in the AEMPS registry, protocol EMH-PRP-2013.

### 4.2. Participants

A total of 58 patients who suffered from knee pain were initially recruited from January to March 2014. Informed concern was obtained from all patients and the study was declared to meet ethical standards by the ethics committee of the Virgen de la Victoria Hospital and AEMPS (EMH-PRP-2013; 29 October 2013).

Patients were aged between 40 and 80 years, had arthritis level I, II or III on the Kellgren–Lawrence scale and pain intensity greater than five on the Visual Analogue Scale (VAS). In case of bilateralism, the more severely-affected knee, based on pain level, was treated. The patient inclusion and exclusion criteria are outlined in Table 1.

According to AEMPS criteria, patients were not included if they were positive for syphilis (RPR), hepatitis and/or HIV, two patients were positive and consequently excluded for the study. Another patient was excluded due to the appearance of a pathology requiring necessary anticoagulation. Platelet count greater than 150.000/mm^3^ was established as inclusion criteria. No patient took anti-aggregants or anticoagulants in the five days prior to blood extraction.

### 4.3. Study Design

After the recruitment phase was completed, 55 patients were eligible for the study according to the inclusion criteria. Patients were assigned using a table of random numbers unknown to patients and to those assessing the outcomes. Twety-eight patients were assigned to group I, to be injected with PRP, and 27 patients to group II, to be treated with HA.

A total of 150 mL of venous blood was taken from all patients using a 16 gauge needle into a bag container holding 21 mL of citrate, phosphate and dextrose (CPD) as an anticoagulant, at the Regional Blood Transfusion Center, which also houses the Umbilical Cord Bank [39]. Group I samples were used to isolate PRP while group II samples were used for a routine blood test. One patient belonging to the PRP group was rejected due to the impossibility of venipuncture. Once the study had begun, one case belonging to HA group was excluded, as she needed arthroscopic surgery due to meniscal tear. Therefore, the study concluded with 27 patients in group I (PRP) and 26 in group II (HA) (Figure 1).

The PRP manufacturing process was performed in the Regional Blood Transfusion Center according to the methods previously described by Marcacci et al. [13]. Briefly, 150 mL of whole blood was distributed into four Falcon test tubes (Thermo Fisher Scientific, Madrid, Spain) that were subjected to double centrifugation and cellular testing, drawing samples off each tube into three opaque syringes for administration to the patient. The fourth tube was reserved for quality-control testing, which included a blood culture for bacteriological testing and a measurement of the concentration of growth factors. Frozen stocks of PRP for each patient were analysed by ProcartaPlex Simplex Human Kit (Affymetrix eBioscience, Vienna, Austria) to quantify growth factor content (HGF, EGF, FGF-2 and PDGF).

### 4.4. Outcome Measures and Follow-up

Patients were treated with three intra-articular injections given at 15 day intervals. Local anaesthesia was not used and the infiltrations were not ultrasound guided. In group I, PRP was administered after thawing at 37 °C for 30 min. The patients assigned to group II were treated with HA in the form of sodium hyaluronate (Adant^®^, Manufactured by Tedec-Meiji Farma, Alcalá de Henares, Madrid, Spain, Molecular Weight: 799.638329 g/mol). Adant^®^, hyaluronic acid, is obtained from cultures of the bacteria Streptococcus zooepidermicus and is supplied as disposable sterile syringes containing 2.5 mL sodium hyaluronate (25 mg). The patient did not know what was being infiltrated, as the syringes for both groups were opaque. Researchers not involved in infiltration and blinded as to which group the patients were assigned carried out patient data collection. The evaluation scales applied at the start of the study were repeated following the third infiltration and after three and six months following the final infiltration, ending the trial. The evaluation scales used were the following: (i) Visual Analogue Scale (VAS), which measured the intensity of the pain experienced by patients [40]; (ii) Knee and Osteoarthritis Outcome System (KOOS), which consisted of five subscales that measure pain, symptoms, function in daily living, function in sport and recreation and knee-related quality of life. This scale was used to determine functional repercussions [41] and (iii) European Quality of Life Scale (EUROQOL), which provided data related to quality of living standards [42].

The oral rescue analgesia allowed in both groups was Acetaminophen 1 g, up to a maximum dosage of 4 g per day. The patient recorded the amount of rescue analgesia needed on a daily basis up to the end of the study. In each group, the procedure’s safety was measured by the appearance of pain or inflammation following infiltration and for delayed adverse effects such as infection, muscular atrophy, profound venous thrombosis, hematoma, tissue hypertrophy or formation of adhesions.

### 4.5. Control Group

A total of 30 patients treated only with drug therapies, excluding intra-articular treatment, were selected as the control group. The treatment consisted in oral administration of pain relievers (metamizol/paracetamol) and anti-inflammatory drugs (ibuprofen 600 mg) with an alternate pattern of 575 mg metamizol/8 h and 1 g Paracetamol/8 h, together with 600 mg ibuprofen rescue if there were no contraindication such as hypertension. Most patients required treatment resume several times over the six-month period of our study, due to aggravation of painful or inflammatory crisis.

The baseline characteristics of the patients within the control group were, Age: (mean + SD): 65.2 ± 4.1; Gender (%): Female (73.3%) and Male (26.6%); Kellgren–Lawrence Knee Osteoarthritis grade (%): I (16.6%), II (27.7%) and III (50%); BMI: (mean ± SD): 31.5 ± 3.4 and Bilateralism (%): Yes (33.3%) and No (66.6%).

### 4.6. Statistical Analysis

A sample size of 26 patients randomly selected from both groups constitutes a power of 85% in order to detect significant differences in the groups of 0.6 units, both in terms of pain and at rest, with a reliability rating of 95%.

In order to measure the effect of infiltration, an analysis of paired samples was carried out between the pain experienced by patients in both groups before and after being infiltrated. Then, the Shapiro–Wilk normality test was applied. In cases in which scores for initial VAS pain and post-intervention VAS pain were normal, the Student’s *t*-test was applied. For the other cases, the Wilcoxon test was used. The same analysis was performed on the KOOS subscales for symptoms, activities, function in sport and recreation and knee-related quality of life after three months and six months, in relation to initial values.

We defined the variable “variation of pain scale” as a difference between the initial and final VAS values, analyzing if there were statistically significant differences between the two groups. For the EUROQOL scale, the variations for each subscale compared to the baseline values were analyzed, establishing the arithmetic difference between both forms, with negative values indicating a worsening, values equal to 0 indicating no change and positive values indicating an improvement.

The relationship between the grade of osteoarthritis and response to treatment was studied by building a multivariate model in which the dependent variable was the variation in the pain score and whose independent variables included the grade of arthritis and the treatment type, controlling for potentially confusing variables such as gender, age and BMI. Also evaluated was whether the bilateral nature of the process or being treated by anesthetists or traumatologists influenced the response to the applied treatment.

## 5. Conclusions

PRP injections proved to be effective in reducing pain and improving patient functionality with an effectiveness pattern comparable to the control treatment (HA). Although no statistically significant differences in pain control were found between treatments, PRP injections proved to improve quality of life of a larger number of patients particularly at three months post-infiltration. Finally, PRP injections seem to be more effective than control HA in patients with less serious arthritis.

## Figures and Tables

**Table 1:** Patient inclusion and exclusion criteria.

Inclusion Criteria	Exclusion Criteria
• Knee Osteoarthritis grade I, II o III Kellgren	• Knee Osteoarthritis grade IV Kellgren
• Knee pain greather than 5 in VAS	• 3 months previous surgery or corticosteroids or hialuronic infiltration
• Age between 40 and 80	• Frontal angular deformity grather than 10°
• Platelet Count > 150.000	• Ipsilateral hip or ankle pathology
• Negative serology to Lues, Hepatitis, HIV	• Knee motion lower than 90° in Flexion or 20° in extension
	• Active anticoagulant therapy
	• Fibromyalgia or chronic fatigue syndrome
	• Liver or haematological disease, infection or neoplasm
	• Pregnancy

VAS: Visual Analogue Scale.

**Table 2:** Baseline characteristics of the patients.

Variable	PRP (*n*: 27)	HA (*n*: 26)	All (*n*: 53)
Age, (mean ± SD)	66.3 ± 8.3	61.5 ± 8.6	63.9 ± 8.7
BMI, (mean ± SD)	29.0 ± 5.5	30.4 ± 4.9	29.7 ± 4.7
Sex, *n* (%)			
Male	12 (44.4)	9 (34.6)	21 (39.6)
Female	15 (55.6)	17 (65.4)	32 (60.4)
Kellgren and Lawrence, *n* (%)			
Grade I	5 (18.5)	2 (7.7)	7 (13.2)
Grade II	10 (37.0)	9 (34.6)	19 (35.9)
Grade III	12 (44.4)	15 (57.7)	27 (50.9)
Bilateralism, *n* (%)			
Yes	15 (55.6)	13 (50.0)	28 (52.8)
No	12 (44.4)	13 (50.0)	25 (47.2)

BMI: body mass index.

**Table 3:** PRP composition. A: Patient Identification number; B: Initial platelet count (10^9^/L); C: Final platelet count after processing (10^9^/L); D: platelet enrichment (C/B); E: Final white blood cell concentration (10^9^/L); F: Final Red Blood cell concentration (10^9^/L).

A	B	C	D	E	F
ID	Initial Platelet	Final Platelet	Platelet Enrichment	Final WBC	Final RBC
2	200	1195	5.98	0.08	0.03
3	196	1245	6.35	0.12	0.04
4	318	1772	5.57	0.59	0.06
5	182	1016	5.58	0.05	0.03
7	227	260	1.15	0.15	0.01
8	262	1575	6.01	0.18	0.08
11	184	1019	5.54	0.08	0.02
14	105	708	6.74	0.05	0.04
15	198	1005	5.08	0.55	0.05
17	215	1623	7.55	0.23	0.06
21	116	580	5.00	0.58	0.06
22	170	1094	6.44	0.26	0.09
25	141	530	3.76	0.02	0.03
26	149	493	3.31	0.07	0.03
28	242	1141	4.71	0.5	0.1
30	206	1031	5.00	0.15	0.04
32	146	614	4.21	0.83	0.09
33	195	1136	5.83	0.07	0.1
34	169	651	3.85	0.7	0.04
37	199	952	4.78	2.58	0.06
38	80	788	9.85	1.26	0.05
39	104	652	6.27	0.17	0.06
43	98	960	9.80	0.85	0.02
48	134	857	6.40	0.11	0.02
51	116	568	4.90	0.62	0.04
52	188	1442	7.67	0.17	0.04
53	234	799	3.41	0.78	0.03

PRP: platelet-rich plasma; WBC: white blood cells; RBC: red blood cells.

**Table 4:** Growth Factor concentration in defrosted PRP.

ID	HGF (pg/mL)	EGF(pg/mL)	FGF-2 (pg/mL)	PDGF-B (pg/mL)
2	207.80	135.79	50.44	1305.94
3	207.77	53.35	64.68	526.93
4	297.85	461.32	208.31	1171.36
5	207.03	321.04	38.94	1841.54
7	361.39	216.79	97.60	1264.04
8	209.25	268.14	59.43	809.05
11	138.76	175.15	<15.23	3984.52
14	175.96	297.75	64.24	1408.47
15	199.92	230.36	<15.23	1366.14
17	193.18	399.96	52.73	941.13
21	113.39	334.12	26.31	550.46
22	183.09	403.14	20.75	619.82
25	138.94	222.93	34.81	1538.01
26	163.33	177.07	43.33	853.68
28	229.19	317.79	75.90	1082.70
30	129.16	316.87	52.80	1569.24
32	252.34	525.75	97.69	2408.36
33	180.15	255.81	59.26	2201.12
34	144.99	254.97	45.26	1004.02
37	212.66	213.43	55.48	1078.99
38	180.16	250.28	60.42	2194.13
39	67.27	194.41	26.49	490.67
43	154.72	205.93	37.99	1679.35
48	170.09	279.99	56.20	1257.45
51	114.53	279.81	46.00	720.43
52	155.75	312.83	57.97	1409.02
53	155.31	187.60	84.47	849.26
Mean	183.11	270.09	60.70	1337.99
Standard Deviation	58.78	100.09	36.51	738.78

ID: Patient Identification number; HGF: Hepatocyte growth factor; EGF: Epidermic growth factor; FGF: Fibroblastic growth factor; PDGF: Platelet derived growth factor.

**Table 5:** EUROQOL Scale Score, comparison between PRP and HA groups.

EUROQOL Pain Scale	Variation	*n* PRP%	PRP	*n* HA	%HA	*n* All	% All
After third infiltration	Worsening	2	7.4	0	0.0	2	3.7
	Similar	20	74.1	17	65.4	37	69.8
	Improvement	5	18.5	9	34.6	14	26.4
*p* = 0.1973	all	27	100.0	26	100.0	53	100.0
3 months after third infiltration	Worsening	1	3.7	3	11.5	4	9.8
	Similar	13	48.1	14	53.8	27	50.9
	Improvement	13	48.1	9	34.6	22	41.5
*p* = 0.4489	all	27	100	26	100.0	53	100.0
6 months after third infiltration	Worsening	2	7.4	4	15.4	6	11.3
	Similar	13	48.1	13	50.0	26	49.0
	Improvement	12	44.4	9	34.6	21	39.6
*p* = 0.6348	all	27	100.0	26	100.0	53	100.0

EQ: EUROQOL (euro quality of life) scale score; PRP: platelet-rich plasma; HA: hyaluronic acid.

**Table 6:** EUROQOL Scale Score in patients treated with oral therapies (Control Group).

EUROQOL Scale	3 Months Post Oral Treatment	6 Months Post Oral Treatment
EQ Mobility	W: 3 (10%); S: 10 (25%); I: 15 (50%)	W: 17 (56.6%); S: 13 (43.3%)
EQ Daily activity	S: 10 (33.3%); I: 20 (66.6%)	W: 20 (66.6%); S: 10 (33.3%)
EQ Personal care	S: 10 (33.3%); I: 20 (66.6%)	W: 20 (66.6%); S: 10 (33.3%)
EQ Pain	S: 15 (50%); I: 15 (50%)	W: 20 (66.6%); S: 10 (33.3%)
EQ Anxiety-depression	S: 10 (33.3%); I: 20 (66.6%)	W: 18 (60%); S: 12 (40%)

W: Worsening; S: Similar and I: Improvement.

**Table 7:** Visual Analogue Scale (VAS) scores for the PRP group and the HA group during the study period regarding baseline values. 95% confidence interval.

VAS Regarding Baseline Values	PRP	HA	*p* Value
50% decrease VAS after third infiltration	15 (55.5%)	15 (57.7%)	1
50% decrease VAS at 3 months after third infiltration	15 (55.5%)	8 (30.7%)	0.227
50% decrease VAS at 6 months after third infiltration	12 (44.4%)	11 (42.3%)	1

**Table 8:** Six months postinfiltration VAS, EQ and KOOS scores according the treating physician.

Scale	Traumatologist (*n* = 36)		Anesthesist (*n* = 17)			Statistical Test
	Mean	SD	Mean	SD	*p* Value	Test
KOOS Pain	62.809	23.582	65.972	24.627	0.661	(a)
KOOS Symptoms	61.706	25.791	70.089	22.993	0.269	(a)
KOOS Daily activity	63.440	26.085	64.314	28.366	0.788	(b)
KOOS Sport	38.194	31.217	39.688	42.248	0.826	(b)
KOOS Quality of Life	39.757	27.188	51.953	35.039	0.179	(a)
EQ Mobility	1.543	0.505	1.600	0.507	0.721	(b)
EQ Personal care	1.486	0.507	1.333	0.617	0.233	(b)
EQ Daily act	1.686	0.471	1.667	0.617	0.800	(b)
EQ Pain	1.943	0.639	2.000	0.535	0.756	(b)
EQ Anxiety–depression	1.486	0.702	1.467	0.743	0.871	(b)
EVA 6 M	4.194	2.638	3.750	2.769	0.583	(a)

VAS: Visual Analogue Scale; EQ: EUROQOL Scale Score; KOOS: Knee injury and Osteoarthritis Outcome Score. (a) Student’s *t*-test for variances homogeneity; (b) *U*-Mann–Whitney–Wilcoxon test. 95% confidence interval.

## Abbreviations

PRP	platelet-rich plasma
HA	hyaluronic acid
VAS	Visual Analogue Scale
KOOS	Knee and Osteoarthritis Outcome System
EUROQOL	European Quality of Life scale
PDGF	platelet-derived growth factor
TGF β	transforming growth factor β
IGF-1	insulin-like growth factor
FGF	fibroblast growth factor
AEMPS	Spanish Agency of Medicines and Medical Devices
CPD	phosphate and dextrose
ASD	daily activities

## References

[1] Lyras D.N., Kazako K., Georgiadis G., Mazis G., Middleton R., Richards S., O’Connor D., Agrogiannis G. (2011). Does a single application of PRP alter the expression of IGF-I in the early phase of tendon healing?. J. Foot Ankle Surg..

[2] Anitua E., Sanchez M., Nurden A.T., Zalduendo M.M., de la Fuente M., Azofra J., Andia I. (2007). Platelet-released growth factors enhance the secretion of hyaluronic acid and induce hepatocyte growth factor production by synovial fibroblasts from arthritic patients. Rheumatology.

[3] Anitua E., Sanchez M., Zalduendo M.M., de la Fuente M., Prado R., Orive G., Andia I. (2009). Fibroblastic response to treatment with different preparations rich in growth factors. Cell Prolif..

[4] Alsousou J., Thompson M., Hulley P., Noble A., Willett K. (2009). The biology of platelet-rich plasma and its application in trauma and orthopaedic surgery. J. Bone Jt. Surg..

[5] Wu C.C., Chen W.H., Zao B., Lai P.L., Lin T.C., Lo H.Y., Shieh Y.-H., Wu C.-H., Deng W.-P. (2011). Regenerative potential of platelet-rich plasma enhanced by collagen in retrieving proinflammatory cytokine-inhibited chondrogenesis. Biomaterials.

[6] Kasemkijwattana C., Menetrey J., Bosch P., Somogyi G., Moreland M.S., Fu F.H., Buranapanitkit B., Watkins S.S., Huard J. (2000). Use of growth factors to improve muscle healing after strain injury. Clin. Orthop. Relat. Res..

[7] Weibrich G., Hansen T., Kleis W., Buch R., Hitzler W.E. (2004). Effect of platelet concentration in platelet-rich plasma on peri-implant bone regeneration. Bone.

[8] Choi B.H., Zhu S.J., Kim B.Y., Huh J.Y., Lee S.H., Jung J.H. (2005). Effect of platelet-rich plasma (PRP) concentration on the viability and proliferation of alveolar bone cells: an in vitro study. Int. J. Oral Maxillofac. Surg..

[9] Kajikawa Y., Morihara T., Sakamoto H., Matsuda K., Oshima Y., Yoshida A. (2008). Platelet-rich plasma enhances the initial mobilization of circulation-derived cells for tendon healing. J. Cell. Physiol..

[10] Sanchez M., Anitua E., Orive G., Mujika I., Andia I. (2009). Platelet-rich therapies in the treatment of orthopaedic sports injuries. Sports Med..

[11] Sampson S., Gerhardt M., Mandelaum B. (2008). Platelet rich plasma injection grafts for musculoskeletal injuries: A review. Curr. Rev. Musculoskelet. Med..

[12] Sampson S., Reed M., Silvers H., Meng M., Mandelbaum B. (2010). Injection of platelet-rich plasma in patients with primary and secondary knee osteoarthritis: A pilot study. Am. J. Phys. Med. Rehabil..

[13] Kon E., Buda R., Filardo G., Di Martino A., Timoncini A., Cenacchi A., Fornasari P.M., Giannini S., Marcacci M. (2010). Platelet-rich plasma: Intra-articular knee injections produced favorable results on degenerative cartilage lesions. Knee Surg. Sports Traumatol. Arthrosc..

[14] Wang-Saegusa A., Cugat R., Ares O., Seijas R., Cuscó X., Garcia-Balletbó M. (2011). Infiltration of plasma rich in growth factors for osteoarthritis of the knee short-term effects on function and quality of life. Arch. Orthop. Trauma Surg..

[15] Ringdahl E., Pandit S. (2011). Treatment of knee osteoarthritis. Am. Fam. Physician.

[16] Zhang W., Moskowitz R.W., NuKi G., Abramson S., Altman R.D., Arden N., Bierma-Zeinstra S., Brandt K.D., Croft P., Doherty M. (2008). OARSI recommendations for the management of hip and knee osteoarthritis, Part II: OARSI Evidence-Based, expert Consensus Guidelines. Osteoarthr. Cartil..

[17] Sanchez M., Fitz N. (2012). A Randomized clinical trial evaluating plasma rich in growth factors (PRGF-Endoret) versus hyaluronic acid in the short-term treatment of symptomatic knee osteoarthritis. Arthrosc. J. Arthrosc. Relat. Surg..

[18] AEMPS (2013). Ministry of Health, Social Services and Equality.

[19] Rodriguez-Merchan E.C. (2013). Intrarticular Injections of Hyaluronic Acid and other drugs in the knee Joint. Hosp. Spec. Surg. J..

[20] Pourcho A.M., Smith J., Wisniewski S.J., Sellon J.L. (2014). Intrarticular platelet-rich plasma injection in the treatment of knee osteoarthritis: Review and recommendations. Am. J. Phys. Med. Rehabil..

[21] Laudy A.B., Bakker E., Rekers M., Moen M.H. (2015). Efficacy of platelet-rich plasma injections in osteoarthritis of the knee: A systematic review and meta-analysis. Br. J. Sports Med..

[22] Patel S., Dhillon M.S., Aggarwal S., Marwaha N. (2013). Treatment with platelet-rich plasma is more effective than placebo for knee osteoarthritis: A prospective, double-blind, randomized trial. Am. J. Sports Med..

[23] Filardo G., Kon E., Pereira-Ruiz M.T., Vaccaro F., Guitaldi R., di Martino A., Cenacchi A., Fornasari P.M., Marcacci M. (2012). Platelet-rich plasma intraarticular injections for cartilage degeneration and osteoarthritis: Singleversus double-spinning Approach. Knee Surg. Sports Traumatol. Arthrosc..

[24] Cerza F., Carni S., Carcangiu A., di Vavo I., Schiavilla V., Pecora A., de Biasi G., Ciuffreda M. (2012). Comparison between hyaluronic acid and platelet-rich plasma, intraarticular infiltration in the treatment of gonarthrosis. Am. J. Sports Med..

[25] Spakova T., Rosocha J., Lacko M., Harvanova D., Gharaibeh A. (2012). Treatment of knee joint osteoarthritis with autologous platelet-rich plasma in comparison with hyaluronic acid. Am. J. Phys. Med. Rehabil..

[26] Wasterlain A.S., Braun H.J., Dragoo J.L. (2012). Contents and formulations of platelet-rich plasma. Oper. Tech Orthop..

[27] Filardo G., Kon E., Buda R., Timoncini A., Di Martino A., Cenacchi A., Fornasari P.M., Giannini S., Marcacci M. (2011). Platelet-rich plasma intraarticular knee injections for the treatment of degenerative cartilage lesions and osteoarthritis. Knee Surg. Sports Traumatol. Arthrosc..

[28] Kon E., Mandelbaum B., Buda R., Filardo G., Delcogliano M., Timoncini A., Fornasari P.M., Giannini S., Marcacci M. (2011). Platelet-rich plasma intraarticular injection versus hyaluronic acid viscosupplementation as treatments for Cartilage pathology: From early degeneration to Osteoarthritis. Arthroscopy.

[29] Roffi A., Filardo G., Assirelli E., Cavallo C., Cenachi A., Fachini A., Grigolo B., Kon E., Mariani E., Pratelli L. (2014). Does platelet-rich plasma freeze-thawing influence growth factor release and their effects on chondrocytes and synoviocytes?. BioMed Res. Int..

[30] Assirelli E., Filardo G., Mariani E., Kon E., Roffi A., Vacaro F., Marcacci M., Facchini A., Pulsatelli L. (2015). Effect of two different preparations of platelet-rich plasma in sinoviocytes. Knee Surg. Sports Traumatol. Arthrosc..

[31] O’Shaughnessey K., Matuska A., Hoeppner J., Farr J., Klaassen M., Kaeding C., Lattermann C., King W., Woodell-May J. (2014). Autologous protein solution prepared from the blood of osteoarthritic patients contains an enhanced profile of anti-inflammatory cytokines and anabolic growth factors. J. Orthop. Res..

[32] Gobbi A., Karnatzikos G., Mahajan V., Malchira S. (2012). Platelet-rich plasma treatment in symptomatic patients with knee osteoarthritis: Preliminary results in a group of active patients. Sports Health.

[33] Sanchez M., Anitua E., Azofra J., Aguirre J.J., Andia I. (2008). Intraarticular injection of an autologous preparation rich in growth factors for the treatment of knee OA: A retrospective cohort study. Clin. Exp. Rheumatol..

[34] Dold A.P., Zywiel M.G., Taylor D.W., Dwyer T., Theodoropoulos J. (2014). Platelet-rich plasma in the management of articular cartilage pathology: A systematic review. Clin. J. Sport Med..

[35] Chang K.V., Hung C.Y., Aliwarga F., Wang T.G., Hag D.S., Cheng W.S. (2014). Comparative effectiveness of platelet-rich plasma injections for treating knee joint cartilage degenerative pathology: A systematic review and meta-analysis. Arch. Phys. Med. Rehabil..

[36] Xie X., Zhang C., Tuan R.S. (2014). Biology of platelet-rich plasma and its clinical application in cartilage repair. Arthritis Res. Ther..

[37] Kayo M., Minako M., Kazuo Y., Tomohiro K., Hiroshi N. (2009). Anti-inflammatory effects of hyaluronan in arthritis therapy: Not just for viscosity. Int. J. Gen. Med..

[38] Russo F., D’Este M., Vadalà G., Cattani C., Papalia R., Alini M., Denaro V. (2016). Platelet rich plasma and hyaluronic acid blend for the treatment of osteoarthritis: Rheological and biological evaluation. PLoS ONE.

[39] Prat I., Hernández-Lamas C., Ortiz M., Sánchez-Gordo F., Vidales I., García G. (2008). Transplantation perspectives in umbilical cord blood banks. Transfus. Apher. Sci..

[40] Flandry F., Hunt J.P., Terry G.C., Hughston J.C. (1991). Analysis of subjective knee complaints using visual analog scales. Am. J. Sports Med..

[41] Roos E.M., Roos H.P., Lohmander L.S., Ekdahl C., Beynnon B.D. (1998). Knee Injury and Osteoarthritis Outcome Score (KOOS) development of a self-administered outcome measure. J. Orthop. Sports Phys. Ther..

[42] Badía X., Herdman M., Montserrat S., Roset M., Segura A. (1999). The Spanish Version of EuroQol: A Description and Its Applications.

